# ASO Author Reflections: Distal Pancreatectomy With and Without Portomesenteric Venous Resection for Pancreatic Adenocarcinoma: A Transatlantic Evaluation of Patients in North America, Germany, Sweden, and The Netherlands (GAPASURG)

**DOI:** 10.1245/s10434-024-16021-1

**Published:** 2024-08-19

**Authors:** Thomas F. Stoop, Simone Augustinus, Bodil Andersson, Poya Ghorbani, Ulrich F. Wellner, Waldemar Uhl, Marc G. Besselink, Henry A. Pitt, Marco Del Chiaro, for Global Audits on Pancreatic Surgery Group (GAPASURG), Bergthor Björnsson, Bobby Tingstedt, Christopher L. Wolfgang, Jens Werner, Karin Johansen, Martijn W. J. Stommel, Matthew H. G. Katz, Michael Ghadimi, Michael G. House, I. Quintus Molenaar, Roeland F. de Wilde, J. Sven D. Mieog, Tobias Keck, Tara M. Mackay, Salvador Rodriguez Franco, Susan van Dieren

**Affiliations:** 1 Division of Surgical Oncology, Department of Surgery, University of Colorado Anschutz Medical Campus, Aurora, CO USA; 2 Amsterdam UMC, Location University of Amsterdam, Department of Surgery, Amsterdam, The Netherlands; 3 Cancer Center Amsterdam, Amsterdam, The Netherlands; 4 Division of Surgery, Department of Clinical Science, Intervention and Technology, Karolinska Institutet at Karolinska University Hospital, Stockholm, Sweden; 5 Department of Clinical Sciences Lund, Surgery, Skåne University Hospital, Lund University, Lund, Sweden; 6 DGAV StuDoQ|Pancreas and Clinic of Surgery, UKSH Campus Lübeck, Lübeck, Germany; 7 Department of Surgery, St. Josef Hospital, Ruhr University Bochum, Bochum, Germany; 8 Rutgers Cancer Institute of New Jersey, New Brunswick, NJ USA; 9 Department of Surgery in Linköping and Department of Biomedical and Clinical Sciences, Linköping University, Linköping, Sweden; 10 Division of Surgical Oncology, Department of Surgery, New York University Medical Center, New York City, NY USA; 11 Department of General, Visceral and Transplant Surgery, University Hospital, LMU Munich, Munich, Germany; 12 Department of Surgery, Radboud University Medical Center, Nijmegen, The Netherlands; 13 Department of Surgical Oncology, The University of Texas MD Anderson Cancer Center, Houston, TX USA; 14 Department of General and Visceral Surgery, University Medical Center Göttingen, Göttingen, Germany; 15 Department of Surgery, Indiana University School of Medicine, Indianapolis, IN USA; 16 Department of Surgery, Regional Academic Cancer Center Utrecht, University Medical Center Utrecht/St. Antonius Ziekenhuis Nieuwegein, Utrecht, The Netherlands; 17 Department of Surgery, Erasmus MC Cancer Institute, University Medical Center, Rotterdam, The Netherlands; 18 Department of Surgery, Leiden University Medical Center, Leiden, The Netherlands

## Past

Portomesenteric venous resection (PVR) has become a standardized procedure in the field of pancreatic cancer surgery, owing to centralization, improved surgical techniques, and increasing use of preoperative chemotherapy.^1,2^ Because approximately two-thirds of the pancreatic adenocarcinomas are located in the pancreatic head, pancreatoduodenectomy (PD) with PVR is mostly performed in case of involvement of the portomesenteric venous axis, associated with similar surgical risks compared with standard PD.^1,3^ However, evidence about pancreatic adenocarcinoma located in the pancreatic body requiring a distal pancreatectomy (DP) combined with PVR is limited, comprising solely single-center series.^4^

## Present

The current international retrospective study aimed to provide insight into the surgical outcome of DP-PVR compared to standard DP (2018–2020) for patients with pancreatic adenocarcinoma, using four audits from North America, Germany, Sweden, and the Netherlands.^5^ A total of 2924 patients were included who underwent DP for pancreatic adenocarcinoma of whom 241 patients (8.2%) underwent DP-PVR. Performing PVR was associated with an increased risk of developing in-hospital/30-day major morbidity, whereas concomitant PVR was not associated with in-hospital/30-day mortality.

## Future

This largest study on DP ± PVR for patients with pancreatic adenocarcinoma demonstrated that DP-PVR is associated with increased morbidity but can be performed safely in terms of mortality. Future studies on patients with pancreatic adenocarcinoma located in the pancreatic body should investigate the short- and long-term surgical and oncological outcomes, comparing DP-PVR with both extended PD-PVR and total pancreatectomy (TP) with PVR.
